# Sleeping To Support? The Interactive Effects of Leader Sleep Quantity and Quality on Leader- and Employee-Reported Support Behaviors

**DOI:** 10.1007/s41542-025-00242-1

**Published:** 2025-10-10

**Authors:** Jordyn J. Leslie, Tori L. Crain, Rebecca M. Brossoit, Leslie B. Hammer, Todd E. Bodner, Cynthia D. Mohr

**Affiliations:** 1 Department of Psychological Science, Weber State University, Ogden, UT USA; 2 Rocky Mountain Center for Occupational and Environmental Health, Salt Lake City, UT USA; 3 Department of Psychology, Portland State University, Portland, OR USA; 4 Department of Psychological Sciences, Rice University, Houston, TX USA; 5 Oregon Institute of Occupational Health Sciences, Oregon Health & Science University, Portland, OR USA

**Keywords:** Sleep, Leaders, Social support, FSSB, Sleep leadership

## Abstract

Although research has documented the relationship between sleep and work outcomes among employees, less research has focused on the role of sleep among workplace leaders. We investigate the link between leader self-reported and actigraphic sleep quantity and outcomes of positive leader support behaviors (i.e., family supportive supervisor behaviors [FSSB], and sleep leadership supportive behaviors [SLSB]) reported by both the leader (*N* = 178) and their direct reports (*N* = 393). Additionally, we examine the interaction between leader sleep quantity and sleep quality indicators (i.e., insomnia symptoms, sleep dissatisfaction, and actigraphic wake after sleep onset [WASO]) on FSSB and SLSB. No main effects were found; however, the results suggest that the relationship between leader sleep and downstream support behaviors is more intricate and nuanced than formerly theorized. Surprisingly, the relationship between leader sleep duration and employee reports of FSSB and SLSB was positive under conditions of high leader insomnia symptoms, yet negative under conditions of low leader insomnia symptoms. A similar pattern emerged for actigraphic total sleep time and employee reports of SLSB which were positive when leaders had increased WASO, yet negative when leaders had lower ratings of WASO. In addition, the relationship between leader sleep duration and leader reports of SLSB was negative when leaders were dissatisfied with their sleep, yet positive when leaders were satisfied. These results inform workplace interventions aimed at promoting leader support behaviors as well as public health campaigns focused on improving sleep health among the general population.

Today’s competitive workplace culture perpetuates the unhealthy belief that successful leaders do not sleep. This is reflected in first-hand accounts from well-known leaders, such as Bill Gates, Steve Jobs, and Margaret Thatcher, who have admitted to neglecting sleep to gain a competitive advantage as a leader (Gates, 2019; Lashbrooke, 2019). These anecdotes are supported by the organizational literature, which suggests that individuals believe getting less sleep leads to career success, such that participants assume successful leaders sleep less than the average worker (Svetieva et al., 2017). Other studies confirm this harmful culture, as the shortest sleep durations and highest fatigue are experienced by leader-level employees as opposed to non-leader employees (e.g., Åkerstedt et al., 2004; Svetieva et al., 2017), indicating that unhealthy sleep beliefs and attitudes are perpetuated by workplace leaders. Taken together, these studies highlight a national concern related to leaders and chronic sleep restriction.

For the average adult, the American Academy of Sleep Medicine and the National Sleep Foundation recommend a minimum of seven hours of sleep per night and high-quality sleep on a regular basis for optimal health and functioning (Hirshkowitz et al., 2015; Watson et al., 2015). A study conducted by the Centers for Disease Control and Prevention uncovered that over one third of Americans (approximately 83.6 million US adults) regularly do not obtain the recommended amount of sleep (Liu et al., 2016). Sleep restriction can be damaging for the worker, the organization, and society, given its major role in depression, anxiety, and emotional exhaustion, as well as organizational outcomes such as engagement, performance, safety, absenteeism, and job satisfaction (e.g., Barnes & Watson, 2019; Litwiller et al., 2017). Unfortunately, research on the consequences of sleep restriction among workplace leaders, specifically, has been largely neglected (Guarana & Barnes, 2017; Svetieva et al., 2017).

Reviews and meta-analyses examining sleep in the workplace demonstrate the field’s narrow focus on employees, as a general category, rather than leaders, as a specific group of employees (e.g., Barnes & Watson, 2019; Henderson & Horan, 2021). Although understanding the relationship between sleep and work outcomes among general employees is important, researchers have called for further examination of the link between sleep and performance among leaders in the workplace (e.g., Gaultney, 2014; Rogers et al., 2019).

Recently, studies have made important contributions to our understanding of the relationship between sleep and social interactions broadly (Gordon et al., 2021), as well as specific leadership behaviors at work. Researchers suggest that variability in abusive leadership (i.e., hostile verbal and nonverbal behavior) can be attributed to poor sleep quality (i.e., feeling rested, ability to fall and stay asleep) (Barnes et al., 2015; Tariq et al., 2020). Additionally, sleep deprivation increases a leader’s tendency to neglect and avoid responsibilities associated with their leadership role (Olsen et al., 2016) and become generally absent when needed by their employees (Bass & Riggio, 2010). These harmful effects of poor sleep are often perceptible to others, as Gaultney (2014) suggests that leaders with inconsistencies between their weekend and weekday sleep duration subsequently receive lower performance ratings from their peers. This subset of literature, however, is overwhelmingly focused on potential negative leadership outcomes of leader sleep.

Less is known regarding the role of sleep in a leader’s ability to engage in *positive* behaviors in the workplace. Few exceptions examine leader sleep and subsequent positive leadership styles such as charismatic leadership (i.e., leaders who engage and inspire followers to believe their group’s mission is extraordinary; Conger et al., 2000) and transformational leadership (i.e., leaders who encourage and empower followers to grow and achieve individual and collective goals; Barnes et al., 2016; Bass & Riggio, 2010; Byrne et al., 2014; Olsen et al., 2016). These studies highlight that as a leader’s sleep worsens, their capacity to engage in positive leadership behaviors declines. This concern is amplified by a recent Gallup poll showing that, post-COVID-19, leaders are more disengaged, burned out, and likely to quit (Barrett, 2023). Leader strain may provide an explanation for another troubling trend from the same poll; since the pandemic, employees have steadily lost faith that their organizations care about them, and their well-being has concurrently declined (Barrett, 2023). Given that leaders are often seen as the face of the organization (Shanock & Eisenberger, 2006), their behavior plays a key role in shaping those perceptions. This may point to a disconnect between what employees want and what leaders are able to provide, raising a critical question for the functioning and sustainability of today’s workplaces: how can we expect leaders to champion well-being when they are “running on empty” themselves?

To this end, it is important to understand how leader sleep is linked with the specific construct of leader social support, and more specifically, support for employee health and well-being. There are two types of support behaviors particularly relevant to this study: family-supportive supervisor behaviors (FSSB) (i.e., actions exhibited by leaders that assist employees in managing family and nonwork demands; Hammer et al., 2009), and sleep leadership supportive behaviors (SLSB) (i.e., actions that aid employees in obtaining more and/or better sleep and reflect concern for employee sleep; Gunia et al., 2015). Both of these positive leader support behaviors target distinct, vital domains of employees’ lives; FSSB is comprised of support behaviors for work and nonwork demands, and SLSB refers to support for employee sleep health. Yet, while the beneficial outcomes of leader support are well-documented (e.g., Hammer et al., 2009; Hammer et al., 2013; Kelloway et al., 2017; Koch & Binneweis, 2015; Las Heras et al., 2015), what drives or prevents leaders from consistently engaging in these behaviors remains largely unexplored (e.g., Crain & Stevens, 2018), especially when considering factors that stem from outside the work domain (e.g., leader health and well-being; Guo et al., 2024). This suggests that we have a limited understanding of how to target, promote, and maintain these supportive behaviors amongst leaders. Thus, this study aims to advance this conversation by examining leader sleep as an antecedent to an intentionally chosen set of positive leader support behaviors in the workplace that address the major domains of an employee’s life, namely work, nonwork, and sleep.

## Contributions

First, we contribute to the organizational literature by focusing on the promotion of positive supportive leader behaviors, which benefit leaders, employees, and organizations alike (e.g., Kelloway et al., 2017; Koch & Binneweis, 2015). FSSB and SLSB were chosen as outcomes for this study because they represent distinct, evidence-based behaviors that benefit the major domains of an employee’s life (work, nonwork, and sleep), have demonstrated organizational value, and remain understudied in terms of antecedents (e.g., Guo et al., 2024). While understanding and mitigating negative leader behaviors is important, focusing solely on prevention is too narrow. By examining positive outcomes, we can learn how to actively promote supportive behaviors. Thus, this study investigates leader sleep as an antecedent to distinct positive leader support behaviors.

The second contribution is our focus on the leader. Although leaders are vital to the improvement of organizational- and employee-level outcomes, antecedents of positive leader support behaviors have largely been overlooked (e.g., Crain & Stevens, 2018; Byrne et al., 2014). Prior work has explored potential antecedents such as gender-related variables (e.g., Sargent, 2022; Braun & Nieberle, 2017) or interventions aimed at improving FSSB and SLSB among supervisors as means to improve employee well-being and safety (e.g., Hammer et al., 2021; Brossoit et al., 2023a), but few studies examine leader-centric antecedents, particularly leaders’ own health. This gap is especially salient given the declining health of workplace leaders in recent years (e.g., Barrett, 2023). Thus, to our knowledge, this is the first study to examine leader sleep as an antecedent to FSSB and SLSB. Moreover, by including both leader and employee reports, we capture a more comprehensive picture of support in the workplace.

Third, we examine the interaction between leader sleep quantity and sleep quality as predictors of downstream leader support behaviors. Research has demonstrated that the correlations between sleep quantity and quality are often small and nonsignificant, adding to the argument that they should be assessed as distinct constructs (e.g., Barnes, 2012; Brossoit et al., 2019; Crain et al., 2018). This study addresses calls to explore the interactive effects of sleep quantity and quality (Barber et al., 2010; Crain et al., 2018), which may clarify how these dimensions jointly shape leader behaviors such as FSSB and SLSB and inform workplace interventions. In addition, unlike past research that assumes sleep is unidimensional, we adopt Buysse’s (2014) multidimensional definition of sleep health, focusing on sleep duration to capture sleep quantity, and sleep satisfaction and insomnia symptoms to reflect aspects of sleep quality. Finally, we use a newer form of interaction graphs coined “tumble graphs” (Bodner, 2016) in place of traditional graphing techniques to depict moderations within dense data regions, avoiding the common error of plotting in sparse regions. This yields a more representative and accurate interpretation of the data.

## Theoretical Rationale

To explain the hypothesized relationship between leader sleep and subsequent support behaviors, we draw from Crain and colleagues (2018) theoretical model which identifies the underlying processes that link the three domains of employees’ lives: work, nonwork, and sleep (WNS). The WNS framework proposes that sleep influences work behaviors, attitudes, and states via energy-based resources: physical energy, defined as the physiological capacity to act (Quinn et al., 2012), and energetic activation, the subjective feeling of vigor and enthusiasm observable in affective outcomes. Sleep contributes to fluctuations in these resources, which in turn shape workplace behaviors such as performance, engagement, and helping (Brief & Weiss, 2002; Crain et al., 2018). Accordingly, we propose that healthy sleep enables leaders to generate the energy-based resources necessary to engage in positive support behaviors at work.

## Hypothesis Development

### The Relationship Between Sleep Quantity and Support Provision of FSSB and SLSB

**Fig. 1:**
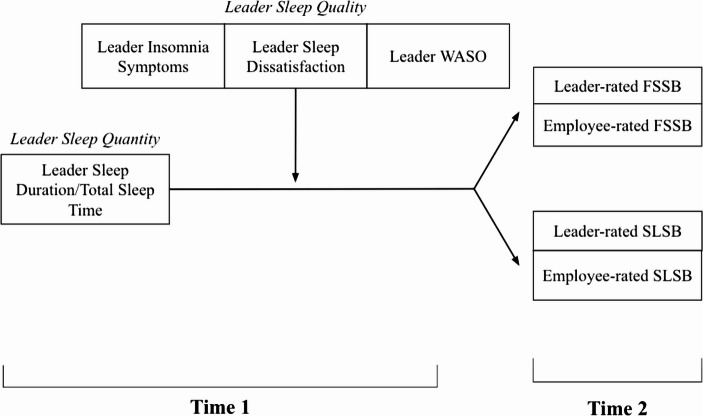
Time lagged moderated model of leader self-report sleep quantity (i.e., sleep duration) and actigraphic sleep quantity (i.e., total sleep time) at Time 1 (i.e., baseline) on FSSB and SLSB at Time 2 (i.e., 9-month), moderated by self-report leader sleep quality (i.e., insomnia symptoms, sleep dissatisfaction) and actigraphic sleep quality (i.e., WASO) at Time 1. Both leaders and their direct employees provided reports of FSSB and SLSB. Control variables (i.e., work schedule, branch of service [Army vs. Air], childcare, eldercare) not shown for parsimony

The construct of FSSB places emphasis on leaders supporting their employees’ nonwork demands, which may include familial obligations, hobbies, educational endeavors, community involvement, time with friends, or other nonwork activities (Kossek et al., 2022). FSSB is conceptualized as domain-specific leader behaviors that enable the employee to be successful in both their work and nonwork lives (Crain & Stevens, 2018; Hammer et al., 2009). Overall, a family-supportive leader is one who “empathizes with the employee’s desire to seek balance between work and nonwork responsibilities” (Thomas & Ganster, 1995, p. 7). It is likely that a leader’s sleep shapes their capacity to provide FSSB, as these behaviors rely heavily on empathy, along with the emotional energy and proactive effort required to recognize and respond to employees’ needs. For instance, a well-rested leader may have the emotional bandwidth to genuinely tune into their employees’ experiences, respond with empathy, and go the extra step to demonstrate FSSB. Research on the antecedents of FSSB has largely overlooked leader health, such as sleep, and has instead emphasized organizational-level influences (e.g., family-supportive policies, workplace culture) and work-domain variables (Guo et al., 2024). This narrow focus neglects nonwork experiences that may significantly shape leader behavior, limiting our knowledge of how support is realistically enacted in the workplace. Our understanding of FSSB precursors is limited, but related research on organizational citizenship behaviors (OCBs), which are discretionary actions beyond formal job duties, offers useful insights (Ellis et al., 2022; Yu et al., 2022). A recent study found that leaders commonly perceive FSSB as outside of their core responsibilities, meaning they choose to engage in it voluntarily (Ellis et al., 2022). This suggests that FSSB demands additional effort beyond traditional job duties, often requiring empathy and emotional intelligence (McKersie et al., 2019; Yu et al., 2022). Research shows that sleep can improve one’s ability to engage in supportive and helping behaviors at work (Åkerstedt et al., 2015; Barnes et al., 2013; Brossoit et al., 2023a; Gordon et al., 2021). Adequate sleep is also critical for empathy (e.g., Guadagni et al., 2017), emotional regulation (e.g., Palmer & Alfano, 2017), and proactivity (e.g., Schmitt et al., 2017), making it easier for leaders to demonstrate these behaviors when well-rested. As such, when leaders have the resources to go above and beyond their required duties, they are more likely to engage in FSSB and more likely to focus their effort beyond core job tasks. We argue that engagement in FSSB depends on sleep-sensitive resources (e.g., empathy), and when such resources are generated due to healthy sleep, leaders are more capable of providing this kind of support. In sum, when a leader gets enough sleep, it is likely easier to demonstrate empathy and proactivity when employee nonwork demands arise and then be more likely to engage in FSSB (Crain et al., 2018) (See Fig. 1).

- Hypothesis 1a-b: Leader sleep quantity at Time 1 will be positively associated with (a) leader and (b) employee reports of FSSB at Time 2.

Although FSSB refers to supervisor behaviors that enable employees to integrate their work and nonwork demands, prior conceptualizations and measures of FSSB do not account for supervisor behaviors directed specifically toward employee sleep. More generally, past literature examining the domains of a working individual’s life has often overlooked sleep; yet, sleep makes up a significant portion of time in a given 24-hour day. Leaders that engage in SLSB demonstrate concern for employee sleep health and help employees meet sleep goals (Adler et al., 2021; Gunia et al., 2015). Similar to the nature of FSSB, SLSB is considered an extra-role behavior, such that the exhibition of SLSB depends on the presence of sleep-generated resources like empathy. We theorize that leaders who obtain sufficient sleep will have the energetic reserves to go above and beyond their core job tasks to demonstrate empathy for their employee’s sleep. Showing care and concern for employee sleep is likely effortful, especially under conditions of poor sleep health. Indeed, sleep research has indicated that sleep restriction impacts effort allocation (Massar et al., 2019), suggesting that leaders with healthy sleep will be more likely to dedicate effort towards caring for employee sleep. In addition, a leader’s own sleep is likely to impact their ability to provide SLSB because if a leader’s sleep is healthy, they should be more aware of how to obtain healthy sleep within their own life and may be more likely to be able to provide that type of support to their employees. If leaders have healthy sleep themselves, they should have both the credibility and knowledge to effectively support sleep in others, thereby fostering greater self-efficacy and motivation, both of which have been meta-analytically tied to increased effort in the workplace (Van Iddekinge et al., 2023). We propose that leaders’ own sleep health is a key antecedent to SLSB, such that when leaders experience sufficient sleep themselves, they may develop greater empathy for the importance of healthy sleep and, in turn, be especially likely to support the sleep health of their employees.

- Hypothesis 2a-b: Leader sleep quantity at Time 1 will be positively associated with (a) leader and (b) employee reports of SLSB at Time 2.

### Interaction Between Sleep Quantity and Sleep Quality

Past literature has typically examined sleep quantity and quality as additive (Barnes, 2012), such that the effects of each sleep dimension are examined individually. The WNS model, however, cites past research in which an interaction effect exists between sleep quantity and quality (Crain et al., 2018), suggesting that the relationship between the constructs could also be multiplicative. Specifically, Barber and colleagues (2010) found that sleep quantity and quality interact to buffer against psychological strain, such that the relationship between sleep quantity and psychological strain is weakened under conditions of high sleep quality. Additionally, Barnes and colleagues (2015) found that the relationship between sleep quantity and daily ego depletion was weakened under conditions of high sleep quality. Due to the novelty of this relationship, researchers have called for further exploration of this effect (Crain et al., 2018). Thus, we expect that when leaders get enough sleep and when they feel rested and satisfied with their sleep, they will engage in more supportive behaviors compared to leaders who obtain insufficient sleep quantity and have poor sleep quality. (See Fig. 1).

- Hypothesis 3a-d: Leader sleep quality at Time 1 will moderate the relationship between leader sleep quantity at Time 1 and (a) employee-reported FSSB, (b) leader-reported FSSB, (c) employee-reported SLSB, and (d) leader-reported SLSB at Time 2, such that the positive relationship between sleep quantity and support behaviors will be enhanced under conditions of high (versus low) sleep quality.

## Method

### Procedure and Participants

Data were collected as part of a larger cluster randomized controlled trial design conducted with service members and their supervisors in the Army and Air National Guard located in one state in the Pacific Northwest region of the United States. The intervention, based on the Total Worker Health™ approach (Anger et al., 2015; Schill & Chosewood, 2013), combined health protection and promotion components. Specifically, supervisors completed a one-hour interactive online training on strategies for supporting employees’ lives outside of work (i.e., health protection). In addition, participants received tailored sleep reports and one-on-one feedback to help them understand their sleep patterns and set improvement goals (i.e., health promotion). For more information on this larger study, please see Hammer et al., 2021. Given the nature of this intervention, we controlled for its effects in the present study’s analyses.

We examined a sample of leaders who were matched with their respective direct employees. Participants were eligible to participate if they worked at least 32 hours per week. Participants were employed primarily in human resources, finance/supply, logistics, and maintenance. Surveys were completed at two time points: Time 1 and approximately 9-months later (Time 2). Participants were also asked to wear actigraphic watches for three weeks at Time 1. As an incentive, participants were offered a gift card for $25 for completing each survey, and $25 for wearing an actigraphy device for 21 days. Employee participants were asked to indicate who their direct leader was in their online survey (i.e., the leader that they report to if they needed to take time off work). The final data set includes employees linked to their respective leader in which leaders had between one and 13 employees matched to them. The final sample sizes for matched leaders and employees were *N* = 178 and *N* = 393, respectively.

Most *leader* participants were white (84.3%), male (80.9%), married (82.6%), were on average 40.8 years old (*SD* = 7.30) and had approximately two children on average (*SD* = 1.4). The most common education level was a college degree (41.6%). Leaders had an average tenure of 5.39 years (*SD* = 5.80), worked an average of 44.83 hours per week (*SD* = 5.31), a regular daytime shift (89.3%), and had approximately six direct reports on average (*SD* = 6.18). Most *employee* participants were white (81.9%), male (74%), married (65.6%), and were on average 35.8 years old (*SD* = 8.86) and had approximately two children on average (*SD* = 1.4). The most common education level was college/technical school with no degree (43.5%). Employees had an average tenure of 4.36 years (*SD* = 5.56), worked an average of 42.37 hours per week (*SD* = 5.0) and a regular daytime shift (81.2%).

### Measures

Scale scores were created for all measures (with the exception of subjective sleep duration and actigraphic sleep measures) using mean imputation if at least 75% of the items were answered per scale.

#### Leader Sleep Quantity

Sleep duration was measured using two items from the Pittsburgh Sleep Quality Index (PSQI; Buysse et al., 1989). The items included, “During the past month, when have you usually gone to bed at night?” and “During the past month, when have you usually gotten up in the morning?”. Leader sleep duration was computed as a difference score between when the leader reported they went to bed and when they woke up.

#### Leader Sleep Quality

Sleep quality was measured using eight total items from the PROMIS Sleep Disturbance scale (Yu et al., 2012), which was separated into two measures based on confirmatory factor analysis results and prior literature (Brossoit et al., 2023b). Four items reflect the sleep dissatisfaction dimension and are reverse-coded. An example item is, “I was satisfied with my sleep” (Cronbach’s α = 0.87). The next four items represent the insomnia symptoms dimension. An example item is, “I had trouble staying asleep” (Cronbach’s α = 0.82). All items were rated on a 5-point scale. As is recommended practice with these measures, scale scores for both dimensions of sleep quality were calculated following the HealthMeasures (2021) scoring system and a t-score transformation metric.

#### Actigraphic Sleep Quantity and Quality

Philips-Respironics Actiwatch 2 devices were used to obtain objective measurements of sleep duration (i.e., total sleep time) and sleep quality (i.e., WASO). Using Actiware 6.0.9, sleep periods were identified by periods of less frequent activity in a given 24-hour period. Total sleep time was calculated by averaging the amount of sleep attained per day in minutes as indicated by the actigraph. Wake after sleep onset (i.e., WASO) refers to the average amount of time in minutes spent awake during each sleeping period. Days were deemed invalid if (1) a watch error occurred, (2) if the watch was removed for four or more hours in a day, and/or (3) if the watch was removed for at least 60 min within 10 min of a sleep period. Participants with fewer than three valid days of actigraphy data from the 21-day period were excluded from analyses.

#### Leader- and Employee-Reported Family-Supportive Supervisor Behaviors (FSSB)

Leaders reported the extent to which they agreed that they exhibited FSSB at Time 2. The short form 4-item FSSB measure (Hammer et al., 2013) was utilized (Cronbach’s α= 0.89). Leaders were asked to respond to four items on a 5-point scale (1 = *strongly disagree*, 5 = *strongly agree*). An example item is, “I make my subordinates feel comfortable talking to me about their conflicts between work and non-work”. Employees who were linked to each leader were also asked to rate the extent to which they agreed that their direct leader exhibited FSSB at Time 2 using the same scale. Employees responded to four items, also rated on a 5-point scale (1 = *strongly disagree*, 5 = *strongly agree*) (Cronbach’s α = 0.95). An example item is, “Your supervisor makes you feel comfortable talking to him/her about your conflicts between work and non-work.”

#### Leader- and Employee-Reported Sleep Leadership Supportive Behaviors (SLSB)

Leaders reported the extent to which they exhibited SLSB at Time 2. SLSB was measured using eight items on a 5-point scale (Gunia et al., 2015) (1 = *never*, 5 = *always*) (Cronbach’s α = 0.88). An example item is, “I encourage my subordinates to get adequate sleep”. Employees were also asked to rate the extent to which their leader exhibited SLSB at Time 2. Employees responded to eight items on a 5-point scale (1 = *never*, 5 = *always*) (Cronbach’s α = 0.94). An example item is, “My supervisor encourages subordinates to get adequate sleep” (Gunia et al., 2015). Please note that the original Gunia et al. (2015) scale included nine items, but we opted to eliminate an item asking about prescription medication use for this study, as we were concerned that the item was sensitive in nature and participants may be worried that it could disqualify them from certain military positions (Hammer et al., 2021).

#### Control variables

A set of control variables for inclusion were selected following Bernerth and colleagues (2018) discussion of the use of statistical control variables. Work schedule (e.g., day, night, variable) and branch of service (i.e., Army Guard versus Air Guard) were included as control variables given past research on job characteristics and sleep (e.g., Åkerstedt, 2003; Whealin et al., 2015). Number of children at home and eldercare were also included as controls given their established relationship with sleep loss (e.g., Khubchandani & Price, 2020; Tienoven et al., 2014). As these data came from a larger randomized control trial which was not of interest to our study, we controlled for the intervention condition to account for the effect of the intervention itself on Time 2 measures given that Time 2 assessments occurred after a group was exposed to the intervention.

## Results

Due to the nested structure of the data (i.e., employees within supervisors), intraclass correlation coefficients (ICCs) were computed and ranged from 0.16 − 0.27, suggesting there is substantial dependency in the outcomes depending on the supervisors that employees were nested under. Accordingly, analyses were conducted using Mplus v 8.4 and multilevel fully-saturated path analyses were specified (Múthen & Múthen, 2018). We ran a series of five moderation models using grand mean centering in which the predictor (i.e., sleep duration), moderators (i.e., sleep dissatisfaction, insomnia symptoms), one outcome (i.e., leader reports of FSSB, employee reports of FSSB, leader reports of SLSB, employee reports of SLSB), and all control variables were included in the model. In addition, we ran four other moderation models in which the actigraphic predictor (i.e., total sleep time), the actigraphic moderator (i.e., WASO), one outcome, and all control variables were included. Table 1 provides descriptive statistics and correlations between study variables. Following analyses, tumble graphs (Bodner, 2016) were constructed in place of traditional graphs. Traditional interaction graphs use arbitrary points (such as + 1 and − 1 SD above and below the mean) which plots the interaction in regions where data can be rather sparse, given that the plotted lines are best fit but may extend beyond regions where data points actually reside. Rather, the use of tumble graphs avoids inaccurate interpretations of the interaction by ensuring that the selected data values reside in populated data regions, making the interaction more representative and interpretable (Bodner, 2016; Rineer et al., 2017).

**Table 1:** Descriptive statistics and correlations among study variables

Variable	*N*	*M*	*SD*	1	2	3	4	5	6	7	8
1. Leader Sleep Duration (T1)	175	7.37	0.99								
2. Leader Insomnia Symptoms (T1)	175	52.47	7.14	.12*	.82						
3. Leader Sleep Dissatisfaction (T1)	175	52.31	7.07	.01	.53**	.87					
4. Leader Actigraphic Total Sleep Time (T1)	153	6.83	0.78	.38**	.19**	.13*					
5. Leader Actigraphic WASO (T1)	153	39.19	12.50	.20**	.28**	.15**					
6. FSSB at T2 (E)	294	4.11	0.90	.05	-.05	-.01	-.02	-.14*	.95		
7. SLSB at T2 (E)	293	2.51	0.48	-.02	-.04	-.03	.02	-.12	.52**	.94	
8. FSSB at T2 (L)	128	4.10	0.49	-.10	-.12*	-.01	-.01	-.06	.02	-.01	.89
9. SLSB at T2 (L)	128	2.70	0.78	-.08	-.14*	-.05	-.06	-.10	-.03	-.03	.22**
10. Branch of Service at T1(E)	393	0.53	0.50	-.21**	-.14**	-.11*	-.27**	.05	.06	-.02	.21**
11. Condition at T1 (E)	393	0.57	0.50	-.07	.52	-.01	-.16**	.11*	-.02	.03	.12*
12. Number of children at T1 (E)	389	1.20	1.21	-.01	-.01	.01	-.03	-.04	-.12	-.02	-.06
13. Eldercare at T1 (E)	393	0.05	0.22	.10*	.10*	.03	.03	-.01	.05	-.05	.06
14. Work Schedule at T1 (E)	393	0.81	0.39	.09	.92	.07	-.01	-.06	-.06	-.05	-.16**
15. Branch of Service at T1 (L)	178	0.56	0.50	-.14**	-.14**	-.11*	-.27**	.05	.06	-.02	.21**
16. Condition at T1 (L)	178	0.48	0.50	.03	.03	-.02	-.16**	-.10	-.03	.02	.10
17. Number of children at T1 (L)	174	1.71	1.40	-.03	-.03	-.06	-.14*	-.01	-.05	.00	-.17**
18. Eldercare at T1 (L)	178	0.05	0.22	.01	.06	.12*	.02	.12*	.02	.01	.01
19. Work Schedule at T1 (L)	178	0.89	0.21	.06	.00	.16**	.05	-.05	-.02	-.04	.04
Variable	9	10	11	12	13	14	15	16	17	18	19
1. Leader Sleep Duration (T1)											
2. Leader Insomnia Symptoms (T1)											
3. Leader Sleep Dissatisfaction (T1)											
4. Leader Actigraphic Total Sleep Time (T1)											
5. Leader Actigraphic WASO (T1)											
6. FSSB at T2 (L)											
7. SLSB at T2 (E)											
8. FSSB at T2 (L)											
9. SLSB at T2 (L)	.88										
10. Branch of Service at T1 (E)	-.09										
11. Condition at T1 (E)	-.06	.16**									
12. Number of children at T1 (E)	-.02	.00	.01								
13. Eldercare at T1 (E)	.01	-.03	-.02	-.05							
14. Work Schedule at T1 (E)	-.06	-.30**	-.05	.05	.02						
15. Branch of Service at T1 (L)	-.09	.10**	.16**	.00	-.03	-.28**					
16. Condition at T1 (L)	-.07	.18**	.97**	.01	-.03	-.05	.18**				
17. Number of children at T1 (L)	-.11	-.07	.16**	-.03	.05	.05	-.07	.15**			
18. Eldercare at T1 (L)	.02	-.05	-.02	.11*	.06	.05	-.05	-.03	-.02		
19. Work Schedule at T1 (L)	-.12*	-.07	.01	.08	-.05	.30**	-.07	.02	-.04	-.01	

### Main Effects

*Hypothesis 1a-b* proposed that Time 1 leader sleep duration would have a positive relationship with leader and employee reports of FSSB at Time 2. There were no significant associations found between leader sleep duration at Time 1 and leader reports of FSSB at Time 2 *(B* = −0.01, *SE* = 0.08, *p* =.95, 95% CI [−0.13, 0.20]) or employee reports of FSSB at Time 2 (*B* = 0.06, *SE* = 0.07, *p* =.41, 95% CI [−0.09, 0.19]), controlling for work schedule, branch of service, elder/child care responsibilities, and the intervention. *Hypothesis 2a-b* proposed that Time 1 leader sleep duration would have a positive relationship with leader and employee reports of SLSB at Time 2. Controlling for work schedule, branch of service, elder/child care responsibilities, and the intervention, there was no significant association between leader sleep duration at Time 1 and leader reports of SLSB at Time 2 (*B* = −0.05, *SE*= 0.11, *p* =.64, 95% CI [−0.27, 0.17]) or employee reports of SLSB at Time 2 (*B* = −0.07, *SE* =0.81, *p* =.41, 95% CI [− 0.02, 0.09]). *Hypotheses 1–2* were not supported (See Table 2).

### Interactions between Sleep Quantity and Quality

**Fig. 2:**
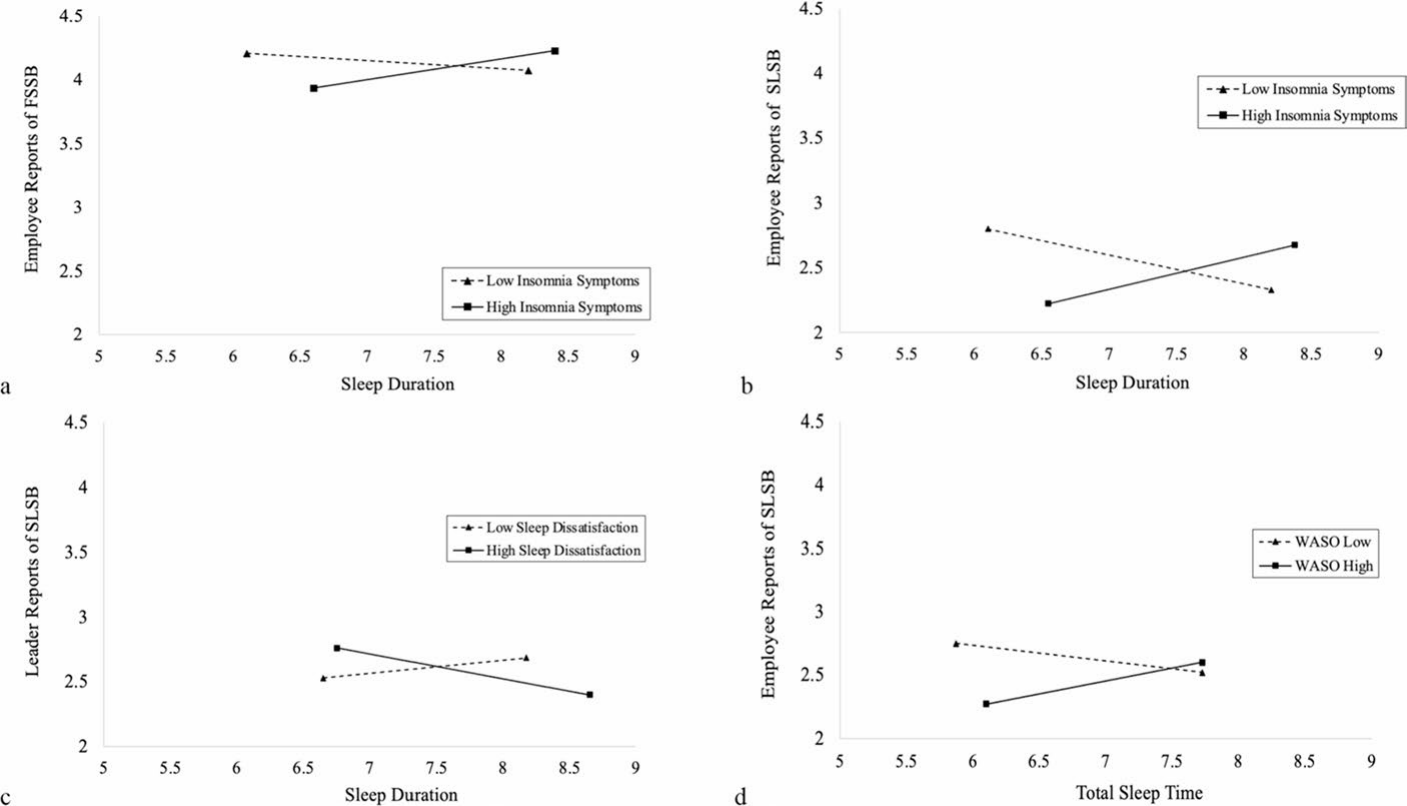
Tumble graphs depicting significant disordinal interactions. Figure 2a depicts the interaction between leader sleep duration and leader insomnia symptoms on employee reports of FSSB. Figure 2b depicts the interaction between leader sleep duration and leader insomnia symptoms on employee reports of SLSB. Figure 2c depicts the interaction between leader sleep duration and leader sleep dissatisfaction on leader reports of SLSB. Figure 2 d depicts the interaction between leader total sleep time and leader WASO on employee reports of SLSB

Results revealed significant interactions between self-reported sleep quantity and sleep quality when insomnia symptoms were considered as a moderator. The relationship between leader sleep duration at Time 1 and employee reports of FSSB at Time 2 (*B* = 0.02, *p* <.05, *pseudo R*^*2*^ = 0.03) as well as employee reports of SLSB at Time 2 (*B* = 0.03, *p* <.01, *pseudo R*^*2*^ = 0.05) was moderated by leader insomnia symptoms at Time 1, controlling for all other variables in the model. Overall, the relationship between leader sleep duration and *employee* reports of FSSB and SLSB was positive under conditions of high leader insomnia symptoms, yet negative under conditions of low leader insomnia symptoms (See Fig. 2a and b). Leader insomnia symptoms did not significantly moderate the relationship between leader sleep duration and *leader* reports of FSSB or SLSB (See Table 2).

**Table 2:** Unstandardized results for hypothesized moderated relationships between leader sleep and positive support behavior outcomes

	Outcomes			
	FSSB(L)	FSSB(E)	SLSB (L)	SLSB (E)
Predictor	b (SE)	b (SE)	b (SE)	b (SE)
Sleep Duration	−0.01 (0.08)	0.06 (0.07)	−0.05 (0.11)	−0.06 (0.08)
Sleep Dissatisfaction	0.01 (0.01)	0.01 (0.01)	0.02 (0.02)	−0.04 (0.07)
Insomnia Symptoms	−0.01 (0.02)	−0.01 (0.01)	−0.02 (0.02)	0.04 (0.05)
Duration x Dissatisfaction	−0.01(0.01)	−0.01 (0.01)	−0.04 (0.02)*	0.01 (0.01)
Duration x Insomnia	0.01 (0.01)	0.02 (0.01)*	0.01(0.02)	0.03 (0.01)**
Actigraphic Total Sleep Time	0.06 (0.10)	0.01 (0.07)	−0.07 (0.13)	0.08 (0.08)
Actigraphic WASO	−0.01 (0.01)	−0.01 (0.00)*	−0.01 (0.01)	−0.01 (0.16)
Total Sleep Time x WASO	0.10 (0.10)	−0.01 (0.01)	0.01 (0.01)	0.02 (0.01)*
Branch of Service	0.10 (0.16)	0.12 (0.14)	−0.24 (0.21)	−0.04 (0.06)
Condition	0.24 (0.13)	−0.04 (0.12)	0.05 (0.19)	0.03 (0.06)
Number of children	−0.07 (0.06)	−0.09 (0.06)	−0.10 (0.07)	−0.05 (0.07)
Eldercare	0.10 (0.37)	0.23 (0.26)	−0.23 (0.55)	−0.04 (0.06)
Work Schedule	−0.26 (0.20)	−0.14 (0.19)	−0.56 (0.45)	−0.07 (0.10)
Pseudo *R*^*2*^	0.02	0.03	0.07	0.05

*b *= Unstandardized Direct Effect. *SE *= Standard Error. E = Employee. L = Leader. FSSB = Family supportive supervisor behaviors, SLSB = Sleep leadership supportive behaviors. Sleep Duration variable is in hours. Branch of service (0 = Army, 1 = Air). Condition (0 = Control, 1 = Treatment). Number of children living at home 3 days a week (0-11). Eldercare (1 = Yes, 0 = No). Work schedule (0 = Other, 1 = Regular Daytime Schedule). Leader* N* = 178, Employee *N* = 393. Employee control variables (i.e., Branch of service, condition, number of children, eldercare, work schedule) were used for employee reported outcomes whereas supervisor variables were used for supervisor/self-reported outcomes. Results reported in this table are those from the supervisor models. Moderator variables were centered around its respective sample mean (grand mean centering). Results did not change significantly when examining employee models. *p < .05, **p < .01, ***p < .00

Additionally, self-reported leader sleep dissatisfaction at Time 1 moderated the relationship between self-reported leader sleep duration at Time 1 and leader reports of SLSB at Time 2 (*B* = −0.04, *p* <.05, *pseudo R*^*2*^ = 0.07), such that the relationship between leader sleep duration and *leader* reports of SLSB was positive under conditions of low leader sleep dissatisfaction, yet negative under conditions of high leader sleep dissatisfaction (See Fig. 2c). Self-reported leader sleep dissatisfaction did not significantly moderate the relationship between leader sleep duration and leader or employee reports of FSSB or employee reports of SLSB (See Table 2).

Finally, the relationship between leader actigraphic total sleep time at Time 1 and employee reports of SLSB at Time 2 were significantly moderated by leader actigraphic WASO at Time 1 (*B* = 0.02, *p* <.05, *pseudo R*^*2*^ = 0.05), such that the relationship between leader total sleep time and employee reports of SLSB was positive under conditions of high WASO, yet negative under conditions of low WASO (See Fig. 2d). WASO did not significantly moderate the relationship between leader total sleep time and leader reports of SLSB or employee or leader reports of FSSB. In summary, results from examined interactions between sleep quantity and quality suggest that *Hypothesis 3a-d* was partially supported.

## Discussion

In this study, we explored the role of self-reported and actigraphic measures of leader sleep quantity and quality on downstream leader support behaviors (i.e., FSSB and SLSB) reported by both leaders and their employees. Hypothesized main effects were not significant, such that sleep quantity was not significantly related to positive support behavior outcomes. Our findings instead suggest that sleep quantity is, by itself, not strongly associated with support behaviors as reported by leaders or their employees. It is only under conditions of certain dimensions of sleep quality that we find significant associations between sleep quantity and positive support behaviors, suggesting that the interaction between sleep quantity and quality is more influential than previously thought. Counter to our hypotheses, leaders with the healthiest sleep were not the most supportive of their employees nonwork lives (i.e., family and sleep). There were also notable differences in the pattern of results depending on the rater of support provision (i.e., leaders or employees).

### Significant Moderated Effects

First, regarding insomnia symptoms as a moderator, we found significant disordinal interactions that were somewhat counterintuitive in light of prior research and theory. For leaders who have high insomnia symptoms, short sleep durations are associated with fewer support behaviors, whereas increases in sleep duration are associated with increases in employee perceptions of familial and sleep support. Conversely, for leaders who have low insomnia symptoms, increases in sleep duration are associated with decreases in employee perceptions of familial and sleep support. These effects are more pronounced for SLSB than FSSB (see Fig. 2a and b). Interestingly, the same pattern of results was found with SLSB when examining the relationship between objective measures of sleep duration (i.e., actigraphic total sleep time) and insomnia symptoms (i.e., actigraphic WASO). For leaders with higher WASO, increases in total sleep time are associated with increases in employee perceptions of sleep support. However, for leaders who have lower rates of WASO, increases in total sleep time are associated with decreases in employee perceptions of sleep support. These effects were found only for *employee*-reported support, rather than self-reported support from leaders.

There are several potential explanations for these findings, each of which are speculative and should be empirically tested in future work. First, in line with our theorizing, leaders who were “poor sleepers” (i.e., high insomnia symptoms and short sleep durations) were perceived as enacting the least supportive behaviors, likely because poor sleep health undermines the empathy required to connect with employees, while also depleting the energetic resources needed to engage in extra-role behaviors like FSSB and SLSB. Counter to our hypotheses, leaders who had low insomnia symptoms and long sleep duration (“good sleepers”) were perceived as engaging in fewer support behaviors. For example, these leaders may not be aware of the value in enacting SLSB because they do not struggle with sleep themselves and may be less likely to empathize with employees who may need this type of support. In other words, the “best” and “worst” sleepers (when considering sleep duration and insomnia symptoms) were perceived as the least supportive. Instead, results suggest that leaders with short sleep duration and low insomnia symptoms and leaders with long sleep duration and high insomnia symptoms are perceived as the most supportive. It is possible that these leaders provide the most support because they *are* able to empathize with employees (Mahsud et al., 2010), due to their personal experience of sleep-related issues (either short sleep or high insomnia symptoms), while still having the energy to provide support from either the absence of insomnia symptoms or adequate sleep duration. These patterns may suggest that supportive leadership emerges most strongly when leaders have both the empathy gained from lived experiences with sleep challenges in combination with the energetic resources to act on that empathy.

Overall, the hypothesized benefits of long sleep duration are only realized under conditions of *poor* sleep quality (i.e., high insomnia symptoms), counter to our expectations. However, it is important to acknowledge that these patterns of results are only found for *employee*-reported perceptions of support. One plausible interpretation is that employees may perceive greater support from leaders who, despite experiencing insomnia symptoms, get enough sleep to maintain the energy needed to be supportive, while their own sleep struggles simultaneously foster empathy that enhances these supportive behaviors. At the same time, perhaps due to their heightened empathy, these leaders may worry that they have not supported their employees enough, which could explain why self-ratings of support follow a somewhat reversed pattern compared to employee-ratings. Alternatively, another speculative mechanism is that leaders with insomnia may be preoccupied with work-related thoughts at night, and obtaining sufficient sleep allows them to replenish resources and address these concerns, which employees may interpret as supportive behaviors. These highly engaged leaders who are dedicated to their work during their nonwork time may be *perceived by their employees* as more supportive even if they struggle with insomnia symptoms, as long as they get enough sleep. In other words, getting more sleep increases resources and allows for the preoccupied leader to address the things they worry about at night. It would be interesting for future work to pinpoint potential causes of insomnia symptoms (e.g., work-related problem-solving pondering; Querstret & Cropley, 2012), which may elucidate these unexpected findings that were only found for employee-reported support. To summarize, the benefits of longer sleep durations appear conditional on higher rates of insomnia symptoms. Perhaps leaders’ experiences of insomnia allow for empathy, and when leaders are actually obtaining enough hours of sleep, employees pick up on these empathetic supportive behaviors. Yet, leaders themselves may still worry that they are not giving enough support in these instances.

Turning our attention to the moderating effects of sleep dissatisfaction, the pattern of results were opposite from the insomnia symptoms moderations. Namely, leaders with low dissatisfaction and long sleep (i.e., “good sleep”) and high dissatisfaction and short sleep (“poor sleep”) reported enacting the most sleep-specific support behaviors. Leaders who obtain adequate sleep and feel rested from their sleep likely have the energy to go the extra mile to support their employee’s sleep needs, whereas leaders who experience poor sleep may develop greater empathy for the challenges of inadequate rest, making them more attuned to employees’ struggles. There may be a dual mechanism at play such that there is an energy-related pathway explaining why this combination of healthy sleep indicators relates to sleep-specific support, and an empathy-related pathway explaining why this combination of unhealthy sleep indicators also relates to sleep-specific support. When sleep is healthy, energetic resources are replenished, which makes it likely that leaders will engage in such behaviors. However, given the nuance of these results, *empathy* towards the issues employees face may play an important role. For example, leaders who have personally experienced sleep difficulties may be more empathetic towards similar struggles among their employees and therefore more likely to recognize and respond to these needs. These results point to the potential of dual energy- and empathy-based pathways, whereby both healthy and unhealthy sleep experiences can foster greater sleep-specific support from leaders.

In addition, leaders with long sleep duration in combination with high sleep dissatisfaction reported the lowest self-reported SLSB toward their employees. Sleeping long hours but not feeling rested or rejuvenated from sleep may be indicative of emotional exhaustion experienced from job-related burnout, which is closely tied to the energetic activation mechanism proposed in the WNS framework (Crain et al., 2018). Burnout, in turn, is often accompanied by a reduced sense of personal accomplishment and efficacy at work (Maslach et al., 2001), which could help explain the disconnect between leaders’ low self-assessments of support and their employees’ perceptions. This may explain why these low *self-reported* support behaviors are not mirrored in their employees’ reports. Disentangling combinations of sleep indicators with related experiences of burnout, or more clinical experiences (e.g., depression, sleep disorders) are important next steps.

Taken together, we found complex and sometimes unexpected interaction effects between leaders’ sleep and their support provision. When insomnia symptoms are examined as a moderator, sleep duration benefits only emerge when sleep quality is poor. Surprisingly, both the “best” and “worst” sleepers were seen as the least supportive by their employees, while those with “mismatched” sleep indicators (short sleep with low insomnia symptoms or long sleep with high insomnia symptoms) were perceived as the most supportive, perhaps due to a balance of empathy and energy. Yet, when dissatisfaction with sleep is examined as a moderator, we found the opposite trend; leaders with both “good” and “poor” sleep reported themselves as being more supportive of their employees’ sleep health, possibly due to greater available empathy and energy. Leaders with long sleep that was not restful or rejuvenating (high dissatisfaction) may be experiencing burnout or mental health concerns. The present findings may indicate that FSSB and SLSB may require substantial emotional and energetic resources, making such behaviors less automatic and more likely to require effort (Crain et al., 2018; DeWall et al., 2008). These findings underscore the nuances of how sleep influences support from leaders and provides numerous opportunities for future research to identify the underlying mechanisms that explain these effects.

Complementing these findings, our use of tumble graphs allows us to make new inferences that have not previously been represented in prior literature. Specifically, because we mapped our interaction graph according to where data were actually present and not just where data might be projected to be (e.g., + 1 and − 1 SD of the moderator), we are able to see that leaders who had lower insomnia symptoms were generally sleeping less than those who had higher insomnia symptoms, which corresponds to the compensatory sleep argument (i.e., people who generally experience insomnia symptoms may need more hours of sleep on average to “make up” for it) (See Fig. 2a and d). In contrast to prior literature and federal recommendations (e.g., Hirshkowitz et al., 2015), these findings support the consideration of different components of sleep quality when conceptualizing and promoting sleep health.

Relatedly, the results contribute to the organizational sleep literature by providing evidence for Buysse’s (2014) multidimensional conceptualization of sleep health, such that there are different components that make up “good” sleep. Historically, organizational research examining sleep has examined sleep quantity and quality as core dimensions (Barnes, 2012). In contrast, Buysse (2014) suggests that sleep health consists of dimensions including sleep duration, sleep satisfaction, sleep efficiency (i.e., ease of falling and staying asleep), alertness/sleepiness, and sleep timing (i.e., placement of sleep within a 24-hour period). Mapping onto this, we examined sleep duration and total sleep time as reflective of sleep quantity, and sleep satisfaction, insomnia symptoms, and WASO as dimensions of sleep quality. We extend Buysse’s (2014) multidimensional conceptualization of sleep health to the organizational sleep literature by teasing apart the nuanced relationships between dimensions of sleep and connecting them to downstream positive leader behavior in the workplace. In addition, organizational scientists know relatively little about how the different components of sleep health may impact important outcomes such as downstream behavior (Crain et al., 2018). Our study shows that the connection between sleep health and downstream workplace behavior may be more complex than previously thought, underscoring the importance of taking a multidimensional approach to measuring sleep health as it may illuminate other unanticipated relationships in future research. On a broader scale, gathering empirical evidence regarding the sleep quantity and sleep quality interaction can inform a new approach for public health campaigns as most campaigns currently emphasize getting at least 7 h of sleep per night (i.e., sleep duration) to maintain adequate functioning (e.g., National Healthy Sleep Awareness Project; American Academy of Sleep Medicine, 2021) and neglect the importance of sleep quality (e.g., efficiency, satisfaction).

### Limitations

There are a number of limitations associated with the present study. Methodologically, in accordance with previous recommendations for studies on sleep and workplace outcomes across time points (e.g., Crain et al., 2018), this study included a 9-month time lag to understand how the relationship between sleep and leader behaviors unfold over time. Although the multi-time point nature of these data are a strength of this study, future research should examine if shorter time lags (e.g., 3-months) or daily fluctuations in sleep quantity and sleep quality have a stronger influence on workplace behavior. Another methodological strength is the inclusion of both leader and employee reports of support provision. On average, leaders and employee agree about the familial and sleep support that the leader provides. However, it is also possible that that leaders may overreport the support they provide, and employees may be inclined to rate their leaders favorably (i.e., social desirability bias). In addition, we were at the relative threshold with our given sample size (178 leaders in the final sample) based on previous recommendations for the type of analyses we conducted (Kline, 2011). Future studies should recruit a larger sample of matched leaders and employees to replicate the results of the present study using more advanced statistical analyses that require greater statistical power. For example, a larger sample would support latent profile analyses (LPA; Smith et al., 2024; Smith & Lee, 2022) which would enable the simultaneous consideration of multiple sleep indicators, helping to capture the complexity and nuance of how distinct patterns of sleep health are associated with different forms of support or motivations for engaging in such behaviors.

Another limitation concerns the generalizability of the findings. While participants held full-time roles in areas like HR and finance, the National Guard context may differ from both civilian workplaces and traditional military settings. For instance, employees may be on-call for high-risk situations (e.g., domestic emergencies, counter-drug efforts), and some roles are safety-sensitive due to contact with machinery or weapons. The sample was also largely composed of white, male, and married individuals. In addition, the leaders in our sample on average received the recommended amount of sleep of about 7 hours per night (*M* = 7.37, *SD* = 0.99), which may limit the generalizability of our findings to populations with more variable or chronically unhealthy sleep patterns. Future research should replicate these findings in other occupational contexts, particularly those with nontraditional schedules (e.g., healthcare workers, restaurant or hotel staff), frequent travel (e.g., construction, athletes, flight attendants), or occupations that may be susceptible to more extreme sleep loss (e.g., nursing). It is also important to explore these research questions using more demographically diverse samples.

Finally, it is important to note that this study did not test the specific mediating mechanisms underlying the relationship between sleep and downstream behavior proposed in the WNS model (i.e., physical energy and energetic activation; Crain et al., 2018), which is an important avenue for future research. Given that our findings did not fully align with our initial theorization, we proposed alternative, but ultimately speculative, explanations. For instance, there may be an empathy-related pathway, in which leaders experiencing poor sleep may be more attuned to or sympathetic toward others facing similar challenges. Mediators worth testing in this context include empathy, burnout, and emotional regulation (Palmer & Alfano, 2017), all of which may shape how leaders respond to others’ well-being needs (Gordon et al., 2021). In addition, there may be an energy-related pathway, whereby well-rested leaders possess greater physical and cognitive resources to offer support. Future studies could test fatigue, perceived alertness, proactivity, cognitive failure (Brossoit et al., 2019), or sleep-related impairment to capture this mechanism. Alternatively, beyond the WNS model, leaders may engage in devaluation of sleep altogether, such that leaders may behave in ways that model or encourage the notion that sacrificing sleep for work is expected or valued (Barnes et al., 2020). In the context of this study, if leaders themselves are not sleeping well and are openly signaling or sharing that with employees, it may be interpreted as an implicit message that sleep is undervalued or dispensable in favor of job demands. This dynamic may be especially relevant in high-demand environments like the military, where sleep is often undervalued and sleep-related disorders are widespread (e.g., Gordon et al., 2021). In sum, this study examined the link between leader sleep and support behaviors but did not directly assess the mediating mechanisms proposed by the WNS model. Empathy-, energy-, and sleep-devaluation pathways remain speculative, highlighting the need for future research to clarify how these processes influence leader support. Explicitly testing these mechanisms is essential to understanding *why* leader sleep affects supportive behaviors.

### Additional Future Directions

Given that this study serves as a steppingstone for uncovering various antecedents to leader support behaviors, particularly those at the leader-level, there are many exciting future directions that researchers can explore. First, future research should utilize the full measure of FSSB (Hammer et al., 2009) to explore how sleep dimensions may differentially be associated with the four components of FSSB: emotional support, instrumental support, role-modeling, and creative work-family management. Taking this direction could lend insight into future interventions aimed at promoting FSSB in the workplace by understanding if and how certain dimensions of FSSB are more or less affected by leader sleep. Secondly, although the present study examines upstream sleep, it would be a particularly interesting avenue for future research to also consider how these hypothesized relationships and work behavior outcomes could impact downstream leader sleep. For example, leaders who feel like they are failing to provide adequate support to their employees may experience large detriments to their sleep due to rumination and guilt. On the other hand, leaders who provide a lot of nonwork support to their employees may feel depleted and therefore, may experience burnout and possible sleep health consequences downstream. Finally, given the research on leaders’ sacrificing of sleep for work (Ruderman et al., 2017) as well as the lack of research on leader health (Barling & Cloutier, 2017) as it relates to their behavior at work, it is critically important to assess how to support leader’s health and well-being. Future research should seek to understand how leaders’ health and well-being may be especially at risk given their unique job tasks, as it may be over and above what non-leader employees experience (Svetieva et al., 2017).

### Practical Implications

Organizations and practitioners should encourage or train employees to be more explicit when asking their leader for nonwork support (e.g., “Can you help me manage my child’s school schedule and my work commitment?”) which may remove the need for leaders to have the necessary sleep-sensitive resources to be acutely attuned to small changes in emotions or ambiguous social cues. In addition, organizations should train leaders on specific tactics for engaging in FSSB or SLSB so that the enactment of such behaviors becomes less contingent on how the leader slept and becomes more automatic with education and practice. Broadly, this study can also inform public health campaigns by shifting the predominant rhetoric given that our findings underscore the importance of sleep quality in downstream behavior. For example, instead of only promoting a bedtime calculator aimed at improving sleep duration, the “7 and up” campaign (American Academy of Sleep Medicine, 2021) could also incorporate information related to sleep hygiene (i.e., sleep habits related to sleep quality such as alcohol or caffeine before bed; Mastin et al., 2006). Additionally, public health campaigns could begin supporting education initiatives about how sleep duration and sleep quality should be addressed holistically (Ohayon et al., 2017).

## Conclusion

The present study investigated the interactive relationship between leader sleep quantity and sleep quality on downstream leader- and employee-reported support behaviors (i.e., family supportive sleep behaviors [FSSBs] and sleep leadership supportive behaviors [SLSB]). There were no main effects, and of the significant interaction effects, most patterns were counter to our hypotheses. Results suggest that the relationships between leader sleep and downstream support behaviors is more complex and nuanced than previously thought, providing exciting opportunities for future research to uncover the mechanisms explaining how and why leaders’ sleep health relates to their engagement in supportive behaviors. Speculatively, our findings may suggest that it is not just the quantity or quality of sleep that matters; our lived experiences with sleep itself may shape how we support others. From these findings, researchers, practitioners, and organizations should prioritize initiatives that promote holistic sleep health among leaders. Public health campaigns should also educate and advocate for the importance of sleep quality in addition to sleep quantity.
